# Married

**Published:** 1867-10

**Authors:** 


***Married.***

In Xenia, Ind., Sept. 22d, 1867, by the Rev. F. W. Keeler, Dr. JUSTIN ROSS, of Williamsport, Ind., to Miss MARIAETTA EGBERT, daughter of Geo. Egbert, M.D., of Xenia, Ind.

